# New Firm—Old Depot

**Published:** 1867-09

**Authors:** 


### NEW FIRM—OLD DEPOT.

This is a world of change; but we suppose our readers like ourselves, regarded Brown's Dental Depot, at the corner of Fourth and Walnut as an exception. It, too, has changed. Dr. Brown has retired, and is succeeded by Messrs. Harvey, Peirce and Cutter. The two latter have been in the establishment for some time, and are consequently well acquainted with its patrons and their wants. And besides, Dr. Peirce, by travel and otherwise, has become very extensively and favorably known by the profession. This *oldest* depot of the West could not have fallen into better hands. The new firm is energetically at work, refittting, and adding new stock, giving evidence of a determination to be surpassed by nothing of the kind East or West. Our readers need not be told to give them a trial.

